# Stage 1 Registered Report: How subtle linguistic cues prevent unethical behaviors

**DOI:** 10.12688/f1000research.20183.4

**Published:** 2020-03-12

**Authors:** Wen Guo, Huanxu Liu, Jingwen Yang, Yuqi Mo, Can Zhong, Yuki Yamada

**Affiliations:** 1 Graduate School of Human-Environment Studies, Kyushu University, Fukuoka, Japan; 2 Faculty of Arts and Science, Kyushu University, Fukuoka, Japan

**Keywords:** cheating, persuasion, attention, moral, self construal, labeling

## Abstract

Different ways of description can easily influence people’s evaluations and behaviors. A previous study by Bryan and colleagues suggested that subtle linguistic differences in ethical reminders can differentially prevent readers’ unethical behavior. The present study aims to replicate the previous finding in the Japanese context, additionally exploring the influence of unfamiliar instruction words that capture participants’ attention. In two experiments, which are planned to be conducted online, participants are asked to make 10 coin-tosses and report the number of “heads” results, indicating the amount of money that they can earn. We will manipulate instructions (“Don’t cheat” vs. “Don’t be a cheater” vs. baseline as a control) for each participant group, including nearly 270 participants (Experiment 1). Next, we will conduct an extended experiment with an additional task in which more attention is directed toward the text (Experiment 2). Through these registered experiments, we examine the credibility of the previous finding that type of instruction affects the occurrence of unethical behaviors.

## Introduction

When people behave dishonestly, they usually downplay the seriousness of the dishonest act (e.g., Monin & Jordan, 2009; Steele, 1988), weakening the link between the dishonesty and one’s self-identity (e.g., Bandura, 1999) to avoid the correspondent inference ( Jones & Nisbett, 1972; Ross, 1977) that one is the kind of person who behaves dishonestly. According to self-concept maintenance theory, individuals in general strive to create and maintain an image of themselves as good and ethical people ( Markus & Wurf, 1987; Mazar *et al.,* 2008).

In general, we believe that highlighting a self-identity word will prevent unethical behaviors to some degree. According to Blasi (1984), a moral person is one for whom moral categories and moral notions are central, essential, and important to self-understanding. Morals cut deeply to the core of what and who such people are as individuals. However, one study revealed that highly constructed self-identities are associated with more unethical behaviors ( Cojuharenco *et al.,* 2012).

Regarding ethical behavior, a moral-character model has been proposed, where moral character consists of motivation, ability, and identity elements ( Cohen & Morse, 2014). Moral identity here refers to being disposed toward valuing morality and wanting to view oneself as a moral person. This disposition should be considered when attempting to understand why people who behave unethically tend to apply a variety of strategies to weaken the behavior–identity link ( Bandura, 1999). The use of “euphemistic labeling” to describe one’s attributes and weaken the link regarding language should also be included in this disposition.

Different ways of description can easily influence people’s evaluation and judgment about something, even if they have a wealth of previously established knowledge ( Fausey & Boroditsky, 2010). For instance, using a transitive verb (agentive description, e.g., “Timberlake ripped the costume”) to describe an accident makes participants significantly more likely to blame the actor compared to the same description with the words changed to an intransitive verb (nonagentive description, e.g., “The costume ripped”). Another study found that, for children aged 5–7 years old, when a noun label was employed to describe a character (e.g., “She is a carrot-eater”) rather than a verbal predicate (e.g., “She eats carrots whenever she can”), their judgment about those characteristics would be more stable over time ( Gelman & Heyman, 1999). The same phenomenon has been demonstrated regarding self-perception ( Walton & Banaji, 2004). It is possible that language has some effect in this category ( Gelman *et al.,* 2000) because when nouns are used to refer to something, one may have a deeper understanding of it, which is noted to “enable inductive inferences” ( Gelman & O’Reilly, 1988).

Once the subtle description is used to refer to oneself, a noun label may have a stronger effect. Bryan *et al.* (2011) found that more people would choose to vote if they heard the words “be a voter” rather than “to vote” on the day before election day. Additionally, research showed that, compared to “helping,” “being a helper” encouraged more children to conduct kind behaviors toward others ( Bryan *et al.,* 2014). However, subsequent research found that although “being a helper” can lead to more kind behaviors initially, once there is a setback, the backlash may also be stronger accordingly ( Foster-Hanson *et al.,* 2018). The reason underlying this phenomenon is as follows: as category labels, nouns bear a strong link to identity and may lead to self-doubt once one fails.

According to Bryan *et al.* (2011), the effect of noun expression comes from a motivation-driven process. When a noun is involved with a positive identity such as “voter” and “helper,” people simply see themselves as voters or helpers and they produce more correlated behaviors; When the noun is involved with undesirable (negative) identities, however, these kind of words should cause people to avoid correlated behaviors.

In social psychology, experiments of priming of unethical behaviors and its subsequent prevention typically involve money or time ( Gino & Mogilner, 2014; Gino & Pierce, 2009; Mogilner & Asker, 2009; Vohs *et al.,* 2006). A mere exposure to money is associated with unethical outcomes ( Kouchaki *et al.,* 2013). In Gino *et al.*’s experiment (2014), participants were asked to complete a scrambled-sentences task using some money-related words or time-related words; results showed that priming time (rather than money) makes people behave more ethically.

In contrast, another experiment by Bryan *et al.* (2013) allowed experimenters to prevent unethical behaviors through semantic priming. They manipulated the task’s instructions by changing the use of verbs (“Don’t cheat”) to noun labels (“Don’t be a cheater”) to inhibit participants from engaging in unethical behaviors. The self-identity related group (“don’t be a cheater”) had significantly lower proportion of unethical cheating behaviors.

In the present study, we aim to replicate Experiment 3 of Bryan *et al.* (2013), for the following reasons:

First, the participants in Experiment 1 in Bryan *et al.* (2013) were asked to think of a number from 1 to 10. If the number was even, they were paid $5; if it was odd, there was no reward. Bryan *et al.* (2013) paid for even numbers because it has been reported that participants typically show a strong bias toward odd numbers in a random number generation task ( Kubovy & Psotka, 1976), but this oddness bias had not been confirmed for betting behaviors. Furthermore, an even or odd number participants think of is just imaginary, occurring in one’s inside world, not an external real event; hence, it is difficult to use it as an index of falsification. An index used for cheating should emphasize that participants’ reports can differ from the fact. Thus, we abandoned the method of Bryan *et al.* (2013) Experiment 1. In their Experiments 2 and 3, they used a coin-tossing task: participants were asked to toss a coin and receive a reward corresponding to the result of their coin flips. We choose this method for our experiment because tossing a coin induces a real external event, which is more objective and operable, and hence it is better than thinking of a number to measure cheating behavior. In addition, compared with Experiment 2 in the original study, which just used two conditions, “cheater” and “cheating”, a baseline group was included in Experiment 3, which made Experiment 3 more complete in its design—an approach we will follow also.

Moreover, we found that the effect size in Experiment 3 was small ( *f* = 0.302 in G*Power (significance level α = 0.05, power level 1-β = 0.95), meaning that Experiment 3 required at least 174 participants; in fact, only 99 people joined the original research. From this, we suppose that the effect size in Experiment 3 was overvalued.

According to the above review, high levels of self-identity and the willingness of individuals to maintain a positive self-view should prevent unethical behaviors. We predict that the self-relevant noun “cheater” will curb cheating behaviors more significantly than the verb “cheating” and the baseline condition (in which there is no reminder in the instruction).

## Methods

### Experiment 1

Our experiment will be conducted online in a private and impersonal way, which means that participants will not meet or be expected to meet the experimenters. We aim to replicate Experiment 3 of Bryan *et al.* (2013), in which there are three conditions: “cheater,” “cheating,” and “baseline”; in the baseline condition, a reminder about cheating will not be mentioned.

***Participants.*** Participants will be users of the Yahoo! Crowdsourcing Service in Japan. Participants are required to meet the *a priori* criterion that they are native Japanese speakers. We plan to conduct a pilot test to determine the shortest time in which one could reasonably participate in the experiment in good faith. This pilot test is detailed in a later section ( *Outlier extraction*). Participants will be excluded if they complete the experiment faster than the pilot test time. Repeat participation will be prevented.

***Procedure.*** At the beginning of the experiment, demographic information of participants’ age and gender will be collected. Online instructions will indicate that a recent controversial article has claimed to report the first scientific evidence for paranormal phenomena ( Bem, 2011); this is the same cover story used in the original study.

We ask the participants to find a coin at home and, while trying to influence the outcome of each toss with their minds, flip the coin 10 times, making it land on “heads” as often as possible. They will be asked to ensure that they are “properly motivated.” For every toss landing on “heads,” they will receive 10 T-points (≈ 10 yen) as a reward. The instructions indicate that participants will receive a reward corresponding to the result of their coin flips. Noting that the average Yahoo! Crowdsourcing user receives two T-points for one task, the reward we set is five times that amount. Even those participants who report a chance level of five “heads” will receive 25 times more points than for a normal task. This reward should be large enough for some participants to engage in cheating behavior.

Participants will be randomly assigned the three conditions. All three conditions will be posted on the crowdsourcing website, and a person who participates in one of the conditions cannot participate in the others. This results in passive randomization. In the “cheater” and “cheating” conditions, the manipulation is embedded in the instructions. We referred to the instructions in the original experiment ( Bryan *et al.,* 2013) and translated it into Japanese, as follows:

NOTE: Please don’t [cheat/be a cheater] and report that one or more of your coin flips landed heads when it really landed tails! Even a small [amount of cheating/number of cheaters] would undermine the study, making it appear that psychokinesis is real.

[嘘をつかない/嘘つきにならない]でください。コイントスの結果，実際にはコインの裏が出たのに表が出たと報告しないでください。

たとえわずかでも[嘘があると/嘘つきがいると]，この研究を台無しにし，念力が実在するように見せてしまいます。

The manipulation is also embedded in the instructions on the next page, where participants log the outcomes of their 10 coin-flips. At the top of the page, a message is displayed in large red words: “嘘をつかないでください/嘘つきにならないでください” This means “PLEASE DON’T [CHEAT/BE A CHEATER]” as in the original experiment ( Bryan *et al.,* 2013).

In the baseline condition, the instructions are the same as above, except that the cheating message is not mentioned.

***Power analysis and sample size.*** Because Experiment 3 of Bryan *et al.* (2013) did not report the effect size, η ^2^, first, we calculated the effect size of the analysis of variance (ANOVA) result from the *F* and *df* values. Bryan *et al.* (2013) reported the statistics of their one-way ANOVA as *F*(2, 96) = 4.38, *p* = .015. Hence, we calculated η ^2^ based on Cohen’s (1973) method, as η ^２^＝.0836. Then, we calculated the effect size, *f*, as follows: *f* = √(η ^2^/(1 – η ^2^) = 0.302. The small sample size may overestimate the effect size so, as a replication convention (e.g., Nitta *et al.,* 2018), we halved the effect size of the original experiment, and used G*Power 3.1.9.3 ( Faul *et al.,* 2009) to conduct a power analysis (i.e., to 0.151). In G*Power, we set the significance level α = 0.05, power level 1-β = 0.95, and effect size *f* = 0.151. According to the conditions of the original experiment, we will divide the participants into three groups. The required total sample size is 681, with 227 participants in each group; therefore, we will try to recruit at least 681 participants, and data collection will not exceed 810 participants. This stopping rule is set because it is difficult for us to limit the number of participants to exactly 681, due to the characteristics of the simultaneous participatory online recruitment system; therefore, we will allow for up to 120% of the required sample size (i.e., 810). If more than 810 people participate in the experiment, we will select the data of the first 810 participants based on the time stamp and use this for the analysis. Also, we set the number of participants (max. 365 males and 445 females) to match the gender distribution of the original study (male: female = .45:.55).

***Data analyses.*** In this study, the dependent variable is the mean number of “heads” reported. In the original experiment, a one-way ANOVA and *t*-test will be performed. Specifically, the ANOVA will be performed for analyzing the main effect of the three groups. A problem in the original study was that the authors did not report adjustments for any significance level in subsequent multiple comparisons. Therefore, in the present study, we will use a one-way ANOVA and Tukey’s method for the multiple comparisons. Additionally, in order to check the cheating in each group, the original study performed one-sample *t*-tests between the mean number of “heads” reported and the chance level (i.e., 50%). These analyses will be performed using jamovi (version 1.0.5). The original results are summarized in Table 1.

**Table 1.** Results of Experiment 3 of Bryan *et al.* (2013).

Analysis types		Reported *p*-value	Degree of freedom	Effect size
Main effect: three groups		.015	96	*f* = 0.302
*t*-test	“cheating” vs “cheater”	.013	96	*d* = 0.71
	“cheater” vs baseline	.004	96	*d* = 0.66
	“cheating” vs baseline	> .80	96	*d* = 0.05
	“cheating” vs “chance”	< .0005	36	*d* = 0.79
	baseline vs “chance”	< .0005	35	*d* = 0.78
	“cheater” vs “chance”	> .30	25	*d* = 0.19

Moreover, as the dependent variable is based on the counts of “heads” reported and that the 10 coin tosses are nested within each participant, a quasi-Poisson or Poisson regression will be used for exploratory analyses. In the (quasi-)Poisson model, the variance is assumed to be the mean multiplied by a dispersion parameter ( Ma *et al.,* 2014). Dispersion parameters with a value greater than one indicate that overdispersion exists; in this case, quasi-Poisson regression will be performed. Thus, which analysis to used depends on the result of variance and the mean of “head “counts. We will first test the original hypothesis. Then, information of gender and age will be added as predictors to establish a regression model.

***Outlier extraction.*** For our online experiment, we will establish a minimum completion time (MCT) for inclusion in the final sample by asking five colleagues who are unfamiliar with this experiment to complete the experiments as fast as possible, then calculating the mean completion time. Specifically, each colleague will perform a coin toss ten times; after each toss they will record the result on the experiment website. This pilot test will not include the attempt to motivate psychokinesis and will measure only the required time of the coin toss and recording. Bryan *et al.* (2013) also used the MCT as an extraction criterion. We will exclude those participants who complete it faster than the MCT, because they may rush through the experiment and fail to complete it in good faith.

### Experiment 2

This experiment is employed as an extended, conceptual replication of Experiment 3 in the original study ( Bryan *et al.,* 2013). Our Experiment 2 is only performed when the results of Experiment 1 successfully replicate those of the original experiment. In the original experiment, the numbers of heads claimed in the “cheater” condition was significantly lower than that in the “cheating” and baseline conditions, but no difference was found between the “cheating” and baseline conditions. Here we cannot easily interpret the non-significant results based on self-identity alone. We aim to test whether lower levels of attention to the instruction in the “cheating” condition reduced the effectiveness of preventing dishonest behaviors in our Experiment 1. Thus, we conduct Experiment 2, adding a “cheating” with task condition in which we use tasks concerning an instruction to ensure that participants’ attention is captured (e.g., Folk *et al.,* 1992; Folk *et al.,* 2002). When we translated the instruction into Japanese, we felt the unfamiliarity of a “cheater” condition in a Japanese language situation. Participants in our experiment may find that the reminder “don’t be a cheater” commands extra attention because of this sense of deviation. Therefore, even if the result of the original experiment is completely reproduced in our Experiment 1, it will not fully support the finding of the original experiment, as the reason for the possible different dishonest behavior rates between the “cheating” and “cheater” conditions in our Experiment 1 may be that the participants in the “cheating” group paid relatively less attention to the instruction; for this reason, “cheating” may have worked weakly as a moral reminder in this condition. Because the experiments are conducted online, it is difficult to ensure that the participants have actually seen and understood the instruction; in addition, it is also possible that the participants ignored the instructions of Experiment 1 due to satisficing, (e.g., Chandler *et al.,* 2014; Oppenheimer *et al.,* 2009; Sasaki & Yamada, 2019), further diminishing the effect of the unattended reminder (i.e., “cheating”). In this Experiment 2 we address these attention-related effects.

Noticeably, the main difference between our Experiment 1 and the original Experiment 3 lies in the different language used in the instruction. Thus, if our Experiment 1 is a successful replication, we will then choose to focus on the expression used in the Japanese instruction, rather than the English instruction of the original Experiment 3.

To support this approach, we conducted a preliminary experiment, asking participants to evaluate their familiarity with certain expressions in Japanese. The expressions “Don’t cheat” and “Don’t be a cheater” were translated into Japanese, and native speakers evaluated their familiarity with them (1: not familiar to 5: very familiar) via an Internet survey on Yahoo! Crowdsourcing. The protocol of this experiment was registered on the Open Science Framework ( Guo *et al.,* 2019). The results showed that the familiarity rating score in the “cheater” condition was significantly lower than that in the “cheating” condition, *t*(64) = 6.73, *p* < .001, Cohen’s *d* = 0.834. Hence, we conjecture that the anticipated difference in the results between the “cheating” and “cheater” conditions in Experiment 1 may partly occur due to differences in attention paid to the instruction, instead of the preservation of a positive self-image proposed by the previous study ( Bryan *et al.,* 2013). This means that part of the effect of the “cheater” condition is due to the unfamiliar expression, which attracts people’s attention then plays a role in preventing them from conducting unethical behavior. See *Extended data* for details about this experiment.

In our Experiment 2, we will manipulate the way in which participants see the instructions to explore the differences between the “cheating” and baseline conditions. Experiment 2 comprises three conditions: “cheating,” “cheating” with task, and baseline. We predict that the “cheating” with task condition will be more effective in curbing unethical behaviors than the “cheating” and baseline conditions, because the task will arouse more attention. While the instruction in the “cheating” condition will be in large red capital letters, this should entail no significant difference compared with baseline.

***Procedure.*** The procedure for Experiment 2 is identical to that of Experiment 1, except for important differences in two aspects. In Experiment 2, we will focus on whether the participants read the instructions as diligently as we expect. First, we will delete the original “cheater” condition and add another “cheating” condition (i.e., “cheating” with task condition). Second, in the “cheating” with task condition, we will add a task page in which participants are asked to choose the exact expression (i.e., “Don’t cheat”) that appeared on the screen from three sample sentences. We will remind participants of this task in advance to ensure they read the instructions carefully.

***Power analysis and participants.*** Because the power analysis of Experiment 2 is the same as in Experiment 1, we intend to recruit participants in the same way as Experiment 1. The minimum completion time will also be established for participants to be included in the final sample. This exclusion standard is similar to that in Experiment 1.

***Data analyses.*** In Experiment 2, the dependent variable is the mean number of “heads” reported. We will still use a one-way ANOVA and Tukey’s method for the multiple comparisons. To check the cheating rate in each group, a one-sample *t*-test between the mean number of “heads” reported and the chance level (50%) will be analyzed. The data of participants who failed to provide the right answer to the attention task will not be used for further analysis. Another analysis by a (quasi-)Poisson regression model will also be performed to explore the contribution factors of cheating counts.

## Study timeline

Currently, the online experiments for participants to conduct the coin-toss task are under construction. After Stage 1 acceptance, our colleagues will be asked to complete the pilot test to calculate the MCT. Then, we will post our experiments on the Yahoo! Crowdsourcing Service to recruit participants. We are supposed to complete the experiments and subsequent analysis within two months.

### Ethical approval and consent to participate

The present study received approval from the psychological research ethics committee of the Faculty of Human-Environment Studies at Kyushu University (approval number: 2019-004). Completion of experiments by participants will be regarded as consent to participate; they will also have the right to withdraw from the experiment at any time without providing a reason. In addition, we will protect participants’ personal information. Because this study will be conducted online, even if participants engage in cheating behaviors, we cannot identify them or meet the participants face-to-face.

## Data availability

### Underlying data

No underlying data are associated with this article.

### Extended data

Open Science Framework: How subtle linguistic cues prevent unethical behaviors, https://doi.org/10.17605/OSF.IO/68FVK ( Guo *et al.,* 2019).

This project contains the following extended data:

- Protocol for the pilot study conducted for Experiment 2.
- Data collected for the pilot study conducted for Experiment 2.

Data are available under the terms of the Creative Commons Attribution 4.0 International license (CC-BY 4.0).
