# Supplementary material for: Stage 1 Registered Report: How subtle linguistic cues prevent unethical behaviors
Source: F1000Res. 2020 Mar 12;8:1482. Originally published 2019 Aug 22. [Version 4] doi: 10.12688/f1000research.20183.4 (PMC7059835; doi:10.12688/f1000research.20183.4)
Supplement: Peer review history

# Peer review: Reviewer response for version 3

**DOI:** 10.5256/f1000research.24963.r60776

**Published:** 2020-03-03

**Authors:** Sergio Cervera-Torres

**Affiliations:** 1 Leibniz-Institute für Wissensmedien (IWM), Tübingen, Germany

The report has very much improved and the authors have answered all my concerns in a reasonable way.

 Perhaps, I have a couple of minor points to outline regarding Experiment 2:

- a) I think that the rationale behind Experiment 2 can be reorganized a bit to understand better the motivation of the "cheating with task" condition. The explanation that you offer to one of my questions (Linguistic issue; point 3) is very clarifying and could be used as a guideline in this regard.

In a similar vein,

- b) If I understand well, both, the "cheating" and "cheating with task" conditions will/should not differ with regard to the baseline. Please, make that point more explicit in the text as you did in your answer.

I confirm that I have read this submission and believe that I have an appropriate level of expertise to confirm that it is of an acceptable scientific standard.

# Response

**Published:** 2020-03-10

**Authors:** Yuki Yamada

**Affiliations:** Kyushu University, Japan

Thank you for your further comments to improve the quality of this manuscript. We will respond to the individual comments below.

*a) I think that the rationale behind Experiment 2 can be reorganized a bit to understand better the motivation of the "cheating with task" condition. The explanation that you offer to one of my questions (Linguistic issue; point 3) is very and could be used as a guideline in this regard.*

**Reply:**

Thank you for your kind suggestion. We have added related sentences to the introduction of Experiment 2 for clarity.

*In a similar vein,*

*b) If I understand well, both, the "cheating" and "cheating with task" conditions will/should not differ with regard to the baseline. Please, make that point more explicit in the text as you did in your answer.*

**Reply:**

Our previous answer was related to one of the possible results that could come about if there is no difference among the three conditions in Experiment 2; we wanted to show how to exclude the influence of attention bias.

In actuality, the pilot experiment showed that there was a significant difference in the familiarity of expressions in the Japanese reminders, so the dishonest behavior rate may be influenced by different levels of attention. For this reason, we predicted that the “cheating” with task condition would induce significantly lower numbers of heads claimed than the “cheating” and baseline conditions. We have clarified this in the manuscript.

# Peer review: Reviewer response for version 2

**DOI:** 10.5256/f1000research.24062.r58686

**Published:** 2020-02-18

**Authors:** Michiko Asano

**Affiliations:** 1 Department of Psychology, College of Contemporary Psychology, Rikkyo University, Saitama, Japan

I feel that the proposal has been substantially improved. The authors have satisfactorily addressed all the issues I had raised. However, I have some minor comments/suggestions on the revised manuscript, which I address below.

**Minor comments:**

1. Data analysis of the “cheating with task” condition in Experiment 2:
  If you are going to delete the data of participants who fail to give the right answer to the attention task, this information should be provided in the main text.
2. The Japanese translation of the instructions in Experiment 1:
  The orders of the [嘘つきにならない/嘘をつかない] (don’t be a cheater/don’t cheat) and [“嘘つきにならないでください/嘘をつかないでください”]（”PLEASE DON’T [BE A CHEATER/CHEAT”）need to be reversed so that they would be consistent with the corresponding English descriptions.
3. I agree with the comment by Dr. Sergio Cervera-Torres on the readability of the introduction. The storyline can be improved.
4. The fourth paragraph of Experiment 2:
  “In our Experiment 2, we manipulated the way in which participants saw….”
  -> “In our Experiment 2, we will manipulate the way in which participants see…”?

I confirm that I have read this submission and believe that I have an appropriate level of expertise to confirm that it is of an acceptable scientific standard.

# Response

**Published:** 2020-02-26

**Authors:** Yuki Yamada

**Affiliations:** Kyushu University, Japan

Thank you very much for approving this manuscript. Here, we will respond to your comments individually.

*I feel that the proposal has been substantially improved. The authors have satisfactorily addressed all the issues I had raised. However, I have some minor comments/suggestions on the revised manuscript, which I address below.*

**Reply:**

We appreciate your positive evaluation and further suggestions. We have further revised the manuscript, taking into account your kind comments.

*1. Data analysis of the “cheating with task” condition in Experiment 2:*

*If you are going to delete the data of participants who fail to give the right answer to the attention task, this information should be provided in the main text.*

**Reply:**

Thank you for your advice. We have added the relevant information in our manuscript as follows: “The data of participants who failed to provide the right answer to the attention task will not be used for further analysis.”

*2. The Japanese translation of the instructions in Experiment 1:*

*The orders of the [* *嘘つきにならない* */* *嘘をつかない* *] (don’t be a cheater/don’t cheat) and [“* *嘘つきにならないでください* */* *嘘をつかないでください* *”]* *（* *”PLEASE DON’T [BE A CHEATER/CHEAT”* *）* *need to be reversed so that they would be consistent with the corresponding English descriptions.*

**Reply:**

Thank you very much for such attention to detail. We have adjusted the order of the reminders in our manuscript to correspond to the original text.

*3. I agree with the comment by Dr. Sergio Cervera-Torres on the readability of the introduction. The storyline can be improved.*

**Reply:**

We have corrected the logical flow of the introduction.

*4. The fourth paragraph of Experiment 2:*

*“In our Experiment 2, we manipulated the way in which participants saw….”*

*-> “In our Experiment 2, we will manipulate the way in which participants see…”?*

**Reply:**

As a registered report, you are correct, this should be written using the future tense. We have changed the tense according to your suggestion.

# Peer review: Reviewer response for version 2

**DOI:** 10.5256/f1000research.24062.r58909

**Published:** 2020-02-06

**Authors:** Sergio Cervera-Torres

**Affiliations:** 1 Leibniz-Institute für Wissensmedien (IWM), Tübingen, Germany

This study aims at replicating, in a Japanese sample, Experiment 3 by Bryan et al. (2013), which investigates whether and how subtle cheating-related ethical reminders (don’t cheat/be a cheater) subsequently prevent cheating behavior. In general, I find the proposal interesting, informative, and worthy to be further conducted. However, I have some concerns/suggestions after carefully reading the report.

**Minor aspects concerning the introduction:**

1. I find the storyline a bit difficult to read. As a suggestion, and if the authors consider it appropriate, I propose reordering some pieces of information. After the first paragraph, I would introduce [According to Blasi (1984)… till (Cojuharenco *et al.*)] followed by [a moral character model… till “euphemistic labeling” regarding language]. Then, [different ways of description…till (Gelman *et al.*]] followed first by the studies regarding ethical behaviour and [According to Bryan *et al.* (2011)…correlated behaviors]. Finally, I would introduce Bryan *et al. *(2013), which is the main study of reference in the current proposal. I think that the motivation of the study could be reinforced beyond the methodological arguments. For example, are there other studies investigating such prevention of unethical behaviour linked to semantic priming? Has this question been investigated in a different cultural setting at all?
2. Please amend the term “decrease” and use “prevent” “reflects less proportion” or something similar. Decrease or increase denotes change. This is not the main point of the study but rather testing the hypothesis that the “cheater” condition will significantly reflect less ratio of cheating behaviour. In the abstract, amend also “no instruction” as control and use baseline control or non-related cheating instruction or something similar. Participants in this group will also have instructions.
3. Does your hypothesis skip the baseline condition for any particular reason? (e.g., “cheater” curbing unethical behavior *more* than both “cheating” and “baseline”).

 

**Methodology section:**

1. I understand that you will use ANOVAS to compare your results with those by Brian *et al.* However, I think that the study will be methodologically more sound if, in addition, you perform an alternative analysis. Considering that your DV is based on counts and that the 10 trials are nested within each participant (therefore probably correlated), I propose a Poisson or quasi-Poisson regression from a generalized estimating equations approach to investigate differences in proportions and Odds Ratio (see Ruiz Fernández, Kastner, Cervera-Torres, Müller, & Gerjets (2019)). You can include gender, age or, another demographic predictor such as job status (working/not working). The analysis can be performed first without such predictors to stick with the original hypothesis and then with the predictors.
2. In the procedure section, I would change “stimulus” I the first lines with “cover story”. In the data analyses section, the term “original” is confusing because it refers to you experiment and Bryans’. Unless you are sure that they didn’t, I suggest stating that [a problem in the original study was that the authors did not report adjustments for any significance level…]

**Linguistic issue:**

 The study by Bryan *et al.* (2013) and the proposed replication relay on the core assumption that instructions compelling self-identity (“don’t be a cheater”) prevent cheating behavior due to “increased” self-identity activation/social-desirability bias. You suspect that the direct translation of the original English instructions “don’t be a cheater” into Japanese might be perceived as rather unfamiliar. In other words, your expected effects might be potentially due to (a) the remainder “cheater” activates self-identity more than the remainder “cheat” and/or (b) the remainder “cheater” promotes extra attentional salience due to unfamiliarity, which, as the authors state, may be problematic to fully support the initial theoretical assumptions. I have some questions in this regard.

  

1. Please, could you clarify whether your preliminary pilot study testing the familiarity of the expressions is based on direct translations from English or analogous expressions in Japanese? It is a bit confusing as it is written in the text “…asking participants to evaluate their familiarity with certain expressions in Japanese. The expressions *Don’t cheat* and *Don’t be a cheater* were translated into Japanese”. In my opinion, using genuine Japanese expressions instead of direct translations should be adequate for a conceptual (cultural) replication.
2. I am wondering if you could find a way to examine whether familiarity predicts/moderates the expected effects. It could be the case that participants grasp the meaning of the expression even if they find it relatively unfamiliar. For example, like a loose idea, a baseline task could be created where participants can rate the familiarity of a series of words/expressions in which the ethical reminders or related words of interest are included. Or, in addition, a multiple-choice task could be designed to reinforce the meaning of the expressions while at the same time potentially reducing unfamiliarity (e.g., in your opinion, which meaning/description do you think that fits better with this expression). If the authors consider that this question is beyond the scope of the study, it would be necessary at least to make this point clear in the discussion.
3. Beyond familiarity, why do you plan to exclude the “cheater” condition in Experiment 2? If you assume that the “cheating” condition captures less attention than the “cheater” one and design a “cheating with task” condition to compensate somehow the attentional bias, should you not compare this new condition with the “cheaters”? I think that you would be able to potentially provide stronger arguments supporting that the attentional bias is less likely to explain the expected findings in Experiment 1.

I confirm that I have read this submission and believe that I have an appropriate level of expertise to confirm that it is of an acceptable scientific standard, however I have significant reservations, as outlined above.

# Response

**Published:** 2020-02-26

**Authors:** Yuki Yamada

**Affiliations:** Kyushu University, Japan

Thank you for your effort and time in reviewing this manuscript. We will respond individually to your comments as follows:

*This study aims at replicating, in a Japanese sample, Experiment 3 by Bryan et al. (2013), which investigates whether and how subtle cheating-related ethical reminders (don’t cheat/be a cheater) subsequently prevent cheating behavior. In general, I find the proposal interesting, informative, and worthy to be further conducted. However, I have some concerns/suggestions after carefully reading the report.*

**Reply:**

We appreciate the use of your valuable time for reviewing this manuscript. We are really pleased that you accurately understand this research and have given it a positive evaluation. We have substantially revised the manuscript to take your kind comments into account.

***Minor aspects concerning the introduction:***

*1. I find the storyline a bit difficult to read. As a suggestion, and if the authors consider it appropriate, I propose reordering some pieces of information. After the first paragraph, I would introduce [According to Blasi (1984)… till (Cojuharenco et al.)] followed by [a moral character model… till “euphemistic labeling” regarding language]. Then, [different ways of description…till (Gelman et al.]] followed first by the studies regarding ethical behaviour and [According to Bryan et al. (2011)…correlated behaviors]. Finally, I would introduce Bryan et al. (2013), which is the main study of reference in the current proposal.*

**Reply:**

Thank you for your insightful comment. We have adjusted the order of the introduction in accordance with your suggestion.

First, we introduced how dishonest acts can be weakened, followed by an explanation of the relationship between dishonest behavior and self-identity based on the words that are being used. We then elaborated how a subtle difference in description affects people’s judgments or behaviors, especially when these words are connected to an individual’s identity. We further introduced Bryan et al.’s experiment, noting its purpose as well as the reasons we wanted to replicate this research.

Regarding the priming effects that you mentioned, as far as we know, similar research that uses semantic priming to prevent unethical behavior has not been conducted. Instead, previous research on priming for unethical behavior has mainly focused on monetary priming (Gino & Mogilner, 2014; Gino & Pierce, 2009; Vohs et al., 2006). A mere exposure to the money construct is associated with unethical outcomes (Kouchaki et al., 2013). Other studies have explored an inhibition effect of time priming on cheating behavior (Gino & Mogilner, 2014; Mogilner & Asker, 2009). In Gino et al.’s experiment (2014), participants were asked to complete a scrambled-sentences task using some money-related words or time-related words; results showed that priming time (rather than money) induces people to behave more ethically. Although the primer stimuli used were different from those in our experiment (time vs. language), both experiments show how to prevent unethical behaviors by encouraging participants to be more conscious of self-identity so as to maintain a positive self-image.

Moreover, we have additional information regarding your question of whether similar studies have been performed in other cultures. Tomer and Eyal also replicated Bryan et al.’s (2013) experiment in Israel (Savir & Gamliel, 2019). All of their participants were native Hebrew speakers. Although the experiments were conducted in a completely different culture, instructions and reminders were still shown in English. They concluded that positive-valence words relating to the self (e.g., “Be an honest person”) have a similar effect on preventing unethical behavior as a negative-valence reminder (“Don’t be a cheater”).

Since the introduction of the experiment results above may help readers to understand the purpose and rationale of our replication better, we decided to substantially reorganize these parts of the manuscript. We apologize that we were unable to introduce all the individual minor changes because they are so many, but please see the text for details. Thank you very much for your suggestion.

*2. Please amend the term “decrease” and use “prevent” “reflects less proportion” or something similar. Decrease or increase denotes change. This is not the main point of the study but rather testing the hypothesis that the “cheater” condition will significantly reflect less ratio of cheating behaviour. In the abstract, amend also “no instruction” as control and use baseline control or non-related cheating instruction or something similar. Participants in this group will also have instructions.*

**Reply:**

Thank you for your suggestions. We amended the words “no instruction” to “baseline” in the abstract. In addition, we replaced the word “decrease” with “prevent” in the introduction as you proposed.

*3. Does your hypothesis skip the baseline condition for any particular reason? (e.g., “cheater” curbing unethical behavior more than both “cheating” and “baseline”).*

**Reply:**

As in our introduction, we mainly discussed how different types of descriptions, especially different expressions of the same meaning in terms of a noun or a verb, influence people’s behaviors. Our hypothesis now covers the baseline condition as follows: “We predict that the self-relevant noun “cheater” will curb cheating behaviors more significantly than the verb “cheating” and the baseline condition (in which there is no reminder in the instruction).” We have added these sentences to the manuscript.

***Methodology section:***

*1. I understand that you will use ANOVAS to compare your results with those by Brian et al. However, I think that the study will be methodologically more sound if, in addition, you perform an alternative analysis. Considering that your DV is based on counts and that the 10 trials are nested within each participant (therefore probably correlated), I propose a Poisson or from a generalized estimating equations approach to investigate differences in proportions and Odds Ratio (see Ruiz Fernández, Kastner, Cervera-Torres, Müller, & Gerjets (2019)). You can include gender, age or, another demographic predictor such as job status (working/not working). The analysis can be performed first without such predictors to and then with the predictors.*

**Reply:**

Thank you for the helpful suggestion. We completely agree with you and decided to add a Poisson or quasi-Poisson regression as exploratory analyses, as the quasi-Poisson regression model is capable of considering overdispersed data, which is a common characteristic in cheating counts. In the Poisson model, the variance is assumed to be the mean multiplied by a dispersion parameter (Ma et al., 2014). The dispersion parameters values that are greater than one indicated that overdispersion exists; in this case, we will use a quasi-Poisson regression for the analysis. Thus, our decision on the analysis will depend on the variance and mean of counts that participants report for “heads.”

In addition, we have two experiments in this study; Experiment 2 will be implemented depending on the results of ANOVA in Experiment 1, not on those of a regression analysis. Thus, the results of the regression analysis will not influence the implementation of Experiment 2. If Experiment 2 is performed, we will also perform a (quasi-)Poisson regression analysis in the results section of Experiment 2. The results of such analyses will be used for discussion in the “General Discussion” part.

We have added these analysis plans and criteria to the revised manuscript.

*2. In the procedure section, I would change “stimulus” I the first lines with “cover story”. In the data analyses section, the term “original” is confusing because it refers to you experiment and Bryans’. Unless you are sure that they didn’t, I suggest stating that [a problem in the original study was that the authors did not report adjustments for any significance level…]*

**Reply:**

Thank you for your suggestion, we have amended the words to “cover story.” If you felt confusion about what the term “original” was referring to, it is likely that other readers may face the same confusion. Hence, we decided to follow your suggestions and have modified the corresponding parts of the revised manuscript.

***Linguistic*** ***issue:***

*The study by Bryan et al. (2013) and the proposed replication relay on the core assumption that instructions compelling self-identity (“don’t be a cheater”) prevent cheating behavior due to “increased” self-identity activation/social-desirability bias. You suspect that the direct translation of the original English instructions “don’t be a cheater” into Japanese might be perceived as rather unfamiliar. In other words, your expected effects might be potentially due to (a) the remainder “cheater” activates self-identity more than the remainder “cheat” and/or (b) the remainder “cheater” promotes extra attentional salience due to unfamiliarity, which, as the authors state, may be problematic to fully support the initial theoretical assumptions. I have some questions in this regard.*

*1. Please, could you clarify whether your preliminary pilot study testing the familiarity of the expressions is based on direct translations from English or analogous expressions in Japanese? It is a bit confusing as it is written in the text “…asking participants to evaluate their familiarity with certain expressions in Japanese. The expressions Don’t cheat and Don’t be a cheater were translated into Japanese”. In my opinion, using genuine Japanese expressions instead of direct translations should be adequate for a conceptual (cultural) replication.*

**Reply:**

Our expression is a direct translation from English. We chose to translate the original English instruction to Japanese directly not because we were hoping to see the difference in familiarity with the two kinds of expressions, but, due to the grammatical features in Japanese, an analogous translation would be difficult. In Japanese, there is a tendency for the subject to be omitted and the corresponding noun connected to that verb to be used less. Thus, if we were to adopt an analogous translation that sounds more familiar, little difference (including whether a verb or a noun is used) would remain between the two kinds of instruction, and the main independent variable in the experiment would disappear.

*2. I am wondering if you could find a way to examine whether familiarity predicts/moderates the expected effects. It could be the case that participants grasp the meaning of the expression even if they find it relatively unfamiliar. For example, like a loose idea, a baseline task could be created where participants can rate the familiarity of a series of words/expressions in which the ethical reminders or related words of interest are included. Or, in addition, a multiple-choice task could be designed to reinforce the meaning of the expressions while at the same time potentially reducing unfamiliarity (e.g., in your opinion, which meaning/description do you think that fits better with this expression). If the authors consider that this question is beyond the scope of the study, it would be necessary at least to make this point clear in the discussion.*

**Reply:**

We do not assume that participants are unable to fully understand the instruction because of unfamiliarity with it. In fact, the Japanese reminder, though not commonly used, can still be understood well by native speakers. We aim to emphasize that, since the unfamiliarity of reminders attracts more attention, the participants are more likely to take note of a less common phrase. Since “don’t cheat” is a very common expression (similar to the slogan “Smoking causes lung cancer” on a cigarette case), participants may ignore it, thus reducing its effect as a moral reminder.

However, your suggestion is very meaningful, and we include a more detailed and clear explanation based on the results of our experiment in the discussion.

*3. Beyond familiarity, why do you plan to exclude the “cheater” condition in Experiment 2? If you assume that the “cheating” condition captures less attention than the “cheater” one and design a “cheating with task” condition to compensate somehow the attentional bias, should you not compare this new condition with the “cheaters”?*

**Reply:**

Experiment 2 is designed and will be conducted only when the results of our Experiment 1 completely replicate the results of the original experiment. In the original experiment, the dishonesty rate for the “don’t be a cheater” condition was significantly lower than that for the “don’t cheat” condition and the baseline, but there was no difference in the dishonesty rate between the “don’t cheat” condition and the baseline. We cannot explain this lack of a significant difference, and we want to analyze the effect of attention (in other words, exclude or confirm whether this happened due to participants’ attention) in our experiment in Japanese. When participants pay enough attention to the reminder “don’t cheat,” is there still no difference in the dishonesty rate between the cheating group and the baseline group? If the results in our Experiment 1 are the same as those in the original experiment, we aim to explore whether lower levels of attention to the cheating condition reduced the effectiveness of preventing dishonest behaviors in our Experiment 1. For this reason, we added a “cheating with task” condition to ensure that the participants actually pay enough attention to the reminder. If there is still no difference in the dishonesty rate for the “cheating with task” condition compared to the baseline, then we can say that the different rate of cheating between “don’t be a cheater” and “don’t cheat” is not because of attention bias.

In this way, your thoughtful and constructive comments have greatly improved our manuscript. We express our sincere gratitude again. We would appreciate it if you approved this manuscript.

**References**

Gino, F., & Mogilner, C. (2014). Time, money, and morality. *Psychological Science, 25*(2), 414-421.

Gino, F., & Pierce, L. (2009). The abundance effect: Unethical behavior in the presence of wealth. *Organizational Behavior and Human Decision Processes, 109*(2), 142-155.

Kouchaki, M., Smith-Crowe, K., Brief, A. P., & Sousa, C. (2013). Seeing green: Mere exposure to money triggers a business decision frame and unethical outcomes. *Organizational Behavior and Human Decision Processes, 121*(1), 53-61.

Ma, L., Yan, X., & Qiao, W. (2014). A quasi-Poisson approach on modeling accident hazard index for urban road segments. Discrete dynamics in nature and society, 2014.

Mazar, N., Amir, O., & Ariely, D. (2008). The dishonesty of honest people: A theory of self-concept maintenance. *Journal of Marketing Research, 45*(6), 633-644.

Mogilner, C., & Aaker, J. (2009). “The time vs. money effect”: Shifting product attitudes and decisions through personal connection. *Journal of Consumer Research, 36*(2), 277-291.

Savir, T., & Gamliel, E. (2019). To be an honest person or not to be a cheater: Replicating the effect of messages relating to the self on unethical behaviour. *International Journal of Psychology, 54*(5), 650-658.

Vohs, K. D., Mead, N. L., & Goode, M. R. (2006). The psychological consequences of money. *Science, 314*(5802), 1154-1156.

# Peer review: Reviewer response for version 1

**DOI:** 10.5256/f1000research.22170.r55680

**Published:** 2019-10-24

**Authors:** Michiko Asano

**Affiliations:** 1 Department of Psychology, College of Contemporary Psychology, Rikkyo University, Saitama, Japan

**Summary**

 This study is designed to examine an interesting topic of whether and how subtle linguistic cues may prevent unethical behaviors. The proposal is generally well written. However, I have some concerns, which I address below.

1. Introduction: It is not clear to me why the authors decided to replicate Bryan *et al*. (2013) ^1^ among many other studies. I understand from the proposal the reason why they chose Experiment 3 of Bryan *et al*. (2013) over Experiment 1 or 2. I also understand that the sample size in Experiment 3 of Bryan *et al*. was not sufficient and thus the results may require replication with a larger sample. However, I do not understand the importance of replicating their results in addressing the research question of whether and how subtle linguistic cues prevent unethical behaviors. The authors should elaborate on this issue to clarify the significance of their study (e.g., “It is important to replicate Bryan *et al*.’s study as it has a significant impact on this field.” or “The protocol of Experiment 3 is useful and applicable to future studies. Therefore, the robustness of their findings should be tested carefully). 
2. Please state in the second to last paragraph of the Introduction that, unlike Experiment 1, Bryan *et al*. (2013) used a coin-tossing task in Experiments 2 and 3. Otherwise, this paragraph is hard to follow.
3. The procedure of Experiment 1 in the current study is not described sufficiently. Please state that the cover story (examination of a paranormal phenomenon) is exactly the same as that of Experiment 3 of Bryan *et al*. (2013).
  It is also necessary to specify how the authors will explain to their participants about the relationship between the coin flips and the amount of reward. Bryan *et al*. (2013) used the following description: "The instructions acknowledged that the laws of probability dictate that people would, on average, make $5, although some would ‘make as much as $10 just by chance’ and others would ‘make as little as $0’”.
  If the procedure is identical to that of Experiment 3 of Bryan *et al*. (2013) except for the use of the Japanese language, stating so would help readers understand the protocol.
4. The authors list the following two points as the reason why they plan to direct participants’ attention toward the ethical reminder in Experiment 2. (a) First, there was no significant difference in the rate of cheating between “don’t cheat” and the baseline (i.e., no ethical reminder) conditions in the study by Bryan *et al*. The authors conjecture that participants in Bryan *et al*.’s did not pay sufficient attention to the ethical reminder. (b) Second, Their preliminary experiment has revealed that the Japanese translation of the expression “don’t be a cheater” (嘘つきにならないで), compared to that of “don’t cheat” (嘘をつかないで), is less familiar to Japanese speakers and thus may attract more attention. Such difference in familiarity may produce results similar to Experiment 3 of Bryan *et al.* (2013), irrespective of whether the expression bears a strong link to self-identity or to the action. To address these concerns, the authors are planning to add “cheating (’don’t cheat’) with task” condition, in which they test whether participants paid attention to the ethical reminder. 
  As for (a), I am not sure whether the lack of difference in cheating rates between “don’t cheat” and the baseline conditions in Bryan *et al*.’s study necessarily means that the participants did not pay attention to the ethical reminder. The cheating rate was significantly lower in “don’t be a cheater” condition than in the “don’t cheat” condition in their study. Doesn’t this mean that the participants paid sufficient attention to the ethical reminder?
  As for (b), I agree with the authors that ethical reminders that attract more attention may prevent unethical behaviors more strongly. However, I do not understand what hypothesis the authors will test using the protocol of Experiment 2. Are they trying to show that it is attention to ethical reminders, rather than the linguistic expression linked to self-identity, that prevents unethical behaviors? If so, how do they interpret the results of Bryan *et al*. (2013)?
5. I do not understand the task of the “cheating with task” condition in Experiment 2. Will the authors use this task to classify participants into those who paid attention to the ethical reminder and those who did not, and analyze the cheating rate separately for these two groups? Or is the task just for bringing participants’ attention to the ethical reminder before the coin-tossing task?

I confirm that I have read this submission and believe that I have an appropriate level of expertise to confirm that it is of an acceptable scientific standard, however I have significant reservations, as outlined above.

## References

[1] : When cheating would make you a cheater: implicating the self prevents unethical behavior. *J Exp Psychol Gen*.2013;142(4) : 10.1037/a0030655 1001-5 10.1037/a0030655 23127418

# Response

**Published:** 2019-12-18

**Authors:** Yuki Yamada

**Affiliations:** Kyushu University, Japan

Thank you very much for taking the time and effort to check our manuscript. we also appreciate your helpful comments. Individual answers are given below.

*1. Introduction: It is not clear to me why the authors decided to replicate Bryan et al. (2013)* *^1^* *among many other studies. I understand from the proposal the reason why they chose Experiment 3 of Bryan et al. (2013) over Experiment 1 or 2. I also understand that the sample size in Experiment 3 of Bryan et al. was not sufficient and thus the results may require replication with a larger sample. However, I do not understand the importance of replicating their results in addressing the research question of whether and how subtle linguistic cues prevent unethical behaviors. The authors should elaborate on this issue to clarify the significance of their study (e.g., “It is important to replicate Bryan et al.’s study as it has a significant impact on this field.” or “The protocol of Experiment 3 is useful and applicable to future studies. Therefore, the robustness of their findings should be tested carefully)* *.*

**Reply:**

 In the present replication study, we are planning to test whether instruction has any effect on the outcome of the experiments. Psychological researchers use instruction before an experiment to explain the aims of the experiment and what participants should do in the experiment. However, up to now it has remained unclear whether these instructions affect the performance of participants. In psychological experiments, one of the problems that affects the results is expectancy effects or participants’ laziness. Laziness is a particularly frequent phenomenon, seeing participants try to finish the experiment as easily as possible just to obtain rewards. Sometimes there is even cheating. There is much evidence suggesting a relationship between reminders and behaviors in the field of social psychology (e.g., Johns, Schmader, & Martens, 2005; Ling, Beenen et al., 2005; Bryan, Master, & Walton, 2014; Bryan, Walton, Rogers, & Dweck, 2011). In particular, Bryan et al. (2013) reported for the first time that a subtle difference in instruction affects cheating. For the reasons above, the results of Bryan et al. are really important for conducting psychological research. Therefore, it is necessary to confirm the reliability of their finding. In fact, another lab has already tried to replicate Bryan et al., although they were not pre-registered (Savir, 2019).

   In Experiment 1 of Bryan et al., participants were asked to think of a number from 1 to 10; if the number was even, they would be paid $5, and otherwise, they would gain no reward. We abandoned this method because it leads to much uncertainty. First, Bryan et al. cited previous research results indicating that participants instructed to generate a random number typically show a strong bias toward odd numbers (Kubovy & Psotka, 1976), so they intentionally paid for even numbers. However, this oddness bias has not been confirmed for betting behaviors. Secondly, thinking of a number is an imaginary event occurring in one’s inside world, not a real external event. Hence, it is suboptimal as an index of falsification, as the index used for cheating should emphasize that the cheating participants’ reports differ from the fact. Therefore, in our experiments, we use the coin-tossing task in Experiments 2 and 3 of Bryan et al. as a method of measuring cheating behavior (falsifying the coin-toss result). Furthermore, compared with Experiment 2 of Bryan et al. which just consisted of two conditions, a baseline group was included in Experiment 3, which made it more complete in design. Therefore, we decided to replicate Experiment 3 of Bryan et al. in our study.

   We amended the introduction to make these points clearer. Thank you for your suggestions, which have helped us clarify the purpose of the present replication.

*2. Please state in the second to last paragraph of the Introduction that, unlike Experiment 1, Bryan et al. (2013) used a coin-tossing task in Experiments 2 and 3. Otherwise, this paragraph is hard to follow.*

**Reply:**

 As a response to this suggestion, we added the following sentences: “In [Bryan et al.’s] Experiments 2 and 3, they used a coin-tossing task: participants were asked to toss a coin and receive a reward corresponding to the result of their coin flips. We choose this method for our experiment because tossing a coin induces a real external event, which is more objective and operable, and hence it is better than thinking of a number to measure cheating behavior.”

*3.The procedure of Experiment 1 in the current study is not described sufficiently. Please state that the cover story (examination of a paranormal phenomenon) is exactly the same as that of Experiment 3 of Bryan et al. (2013).*

*It is also necessary to specify how the authors will explain to their participants about the relationship between the coin flips and the amount of reward.* *Bryan et al. (2013) used the following description: "The instructions acknowledged that the laws of probability dictate that people would, on average, make $5, although some would ‘make as much as $10 just by chance’ and others would ‘make as little as $0’”.*

*If the procedure is identical to that of Experiment 3 of Bryan et al. (2013) except for the use of the Japanese language, stating so would help readers understand the protocol.*

**Reply:**

 In the original experiment, Bryan et al. referred to the article by Bem (2011, which had received considerable media attention) to describe a recent discovery of evidence for paranormal phenomena. We will use the same article for our instruction. As the details of the paranormal article are not given in Bryan et al.’s paper, we will abstract the content of Bem’s article and translate it into Japanese and add this part to our instructions.

   As for the relationship between the coin flips and reward, we will instruct participants that they will receive a reward corresponding to the result of their coin flips. In the Yahoo! Crowdsourcing Service, we gave a reward of about 5 times the average, to encourage cheating. However, the rewards that each person ultimately gets still depend on the result of their coin flips.

*4,* *The authors list the following two points as the reason why they plan to direct participants’ attention toward the ethical reminder in Experiment 2. (a) First, there was no significant difference in the rate of cheating between “don’t cheat” and the baseline (i.e., no ethical reminder) conditions in the study by Bryan et al. The authors conjecture that participants in Bryan et al.’s did not pay sufficient attention to the ethical reminder. (b) Second, Their preliminary experiment has revealed that the Japanese translation of the expression “don’t be a cheater” (* *嘘つきにならないで* *), compared to that of “don’t cheat” (* *嘘をつかないで* *), is less familiar to Japanese speakers and thus may attract more attention. Such difference in familiarity may produce results similar to Experiment 3 of Bryan et al. (2013), irrespective of whether the expression bears a strong link to self-identity or to the action. To address these concerns, the authors are planning to add “cheating (’don’t cheat’) with task” condition, in which they test whether participants paid attention to the ethical reminder.*

*As for (a), I am not sure whether the lack of difference in cheating rates between “don’t cheat” and the baseline conditions in Bryan et al.’s study necessarily means that the participants did not pay attention to the ethical reminder. The cheating rate was significantly lower in “don’t be a cheater” condition than in the “don’t cheat” condition in their study. Doesn’t this mean that the participants paid sufficient attention to the ethical reminder?*

*As for (b), I agree with the authors that ethical reminders that attract more attention may prevent unethical behaviors more strongly. However, I do not understand what hypothesis the authors will test using the protocol of Experiment 2. Are they trying to show that it is attention to ethical reminders, rather than the linguistic expression linked to self-identity, that prevents unethical behaviors? If so, how do they interpret the results of Bryan et al. (2013)?*

**Reply:**

 Thank you for your comments. We will answer your questions one by one.

*As for (a), I am not sure whether the lack of difference in cheating rates between “don’t cheat” and the baseline conditions in Bryan et al.’s study necessarily means that the participants did not pay attention to the ethical reminder. The cheating rate was significantly lower in “don’t be a cheater” condition than in the “don’t cheat” condition in their study. Doesn’t this mean that the participants paid sufficient attention to the ethical reminder?*

   Possibly our description in the draft was not clear enough. In fact, we did not discuss participants’ attention in the original experiment. However, in our replication experiment, we realized that the attention of the participants to the reminder may become a problem, and aimed to explore this. As your comment reflects, the existing evidence is not sufficient to address this problem, nor, of course, can our experiments clarify the problem of attention among the participants in the original experiment. Based on result of our Experiment 2, participants’ attention to reminders in the original experiment will be discussed in the *General discussion* section of the paper (which is not included in the Stage 1 manuscript). Hence, we removed reason (a) in our draft and instead described the reason why our Experiment 2 had to highlight the issue of attention.

   We made the following changes to the manuscript: “When we translated the instruction into Japanese, we felt the unfamiliarity of a “cheater” condition in a Japanese language situation. Participants in our experiment may find that the reminder “not to be a cheater” captures extra attention because of this sense of deviation. Therefore, even if the result of the original experiment is completely reproduced in our Experiment 1, it will not fully support the finding of the original experiment, as the reason for the possible different dishonest behavior rates between the “cheating” and “cheater” conditions in our Experiment 1 may be that the participants in the “cheating” group paid relatively less attention to the instruction, so that “cheating” weakly worked as a moral reminder in this condition. Because the experiments are conducted online, it is difficult to ensure that the participants have actually seen and understood the instruction; in addition, it is also possible that the participants ignored the instruction of our Experiment 1 due to satisficing, (e.g., Chandler et al., 2014; Oppenheimer et al., 2009; Sasaki & Yamada, 2019), further diminishing the effect of the unattended reminder (i.e., “cheating”).”

*As for (b), I agree with the authors that ethical reminders that attract more attention may prevent unethical behaviors more strongly. However, I do not understand what hypothesis the authors will test using the protocol of Experiment 2. Are they trying to show that it is attention to ethical reminders, rather than the linguistic expression linked to self-identity, that prevents unethical behaviors? If so, how do they interpret the results of Bryan et al. (2013)?*

   We will mainly discuss reason (b). We hypothesized that there is a difference in the dishonest behavior rate between the “cheating” with task condition and baseline in Experiment 2 based on the premise of the results of the preliminary experiment (i.e., the familiar expressions used in the two reminders are certainly different).

   In order to convey the relationship between the preliminary experiment and the hypothesis more clearly, we made the following amendments to the manuscript: “Our Experiment 2 will only be performed when the results of Experiment 1 successfully replicate those of the original experiment. We will conduct Experiment 2, adding a “cheating” condition in which we use tasks concerning an instruction to ensure that participants’ attention is captured.” In this way, we want to find out if our experiments really support (or do not support) the results of the original experiment.

*5, I do not understand the task of the “cheating with task” condition in Experiment 2. Will the authors use this task to classify participants into those who paid attention to the ethical reminder and those who did not, and analyze the cheating rate separately for these two groups? Or is the task just for bringing participants’ attention to the ethical reminder before the coin-tossing task?*

**Reply:**

 We intend to use this task only to make sure that participants pay enough attention to the ethical reminder. We will delete the data of participants who fail to give the right answer to the attention task.

***References***

 Bem, D. J. (2011). Feeling the future: experimental evidence for anomalous retroactive influences on cognition and affect. *Journal of personality and social psychology, 100*(3), 407.

 Bryan, C. J., Master, A., & Walton, G. M. (2014). “Helping” versus “being a helper”: Invoking the self to increase helping in young children. *Child Development*, *85*(5), 1836-1842.

 Bryan, C. J., Walton, G. M., Rogers, T., & Dweck, C. S. (2011). Motivating voter turnout by invoking the self. *Proceedings of the National Academy of Sciences*, *108*(31), 12653-12656.

 Johns, M., Schmader, T., & Martens, A. (2005). Knowing is half the battle: Teaching stereotype threat as a means of improving women's math performance. *Psychological Science*, *16*(3), 175-179.

 Ling, K., Beenen, G., Ludford, P., Wang, X., Chang, K., Li, X., ... & Resnick, P. (2005). Using social psychology to motivate contributions to online communities. *Journal of Computer* *‐* *Mediated Communication*, *10*(4), 00-00.

 Savir, T., & Gamliel, E. (2019). To be an honest person or not to be a cheater: Replicating the effect of messages relating to the self on unethical behaviour. *International Journal of Psychology, 54*(5), 650-658.

 In this way, your thoughtful and constructive comments have greatly improved our manuscript. We express our sincere gratitude again. We would appreciate it if you approved this manuscript.
